# Exercise serum regulates uric acid transporters in normal rat kidney cells

**DOI:** 10.1038/s41598-022-22570-w

**Published:** 2022-10-27

**Authors:** Zhongye Jiang, Jianmin Cao, Hao Su, Hui Cao, Zeyuan Sun, Haoze Jiang, Yanjun Fan

**Affiliations:** 1 Sport Biochemistry Department, Sport Science College, Beijing Sport University, Beijing, China; 2 North China Electric Power University, Beijing, China

**Keywords:** Kidney diseases, Biochemistry

## Abstract

Hyperuricemia (HUA) refers to a physiological condition of high serum uric acid (SUA) level in the body, which may cause an increased risk of several chronic diseases. The kidney’s impaired uric acid (UA) metabolism is an important reason for HUA. In this study, we tested the hypothesis that circulating factors produced during exercise regulate the expression of ABCC4, ABCG2, URAT1, and GLUT9 in normal rat kidneys and normal rat kidney cells (NRK-52E) and their relationship with NF-κB and NRF-2. NRK-52E cells were separately cultured by serum from 10 healthy SD rats who did not exercise (CON) and 10 healthy SD rats who did aerobic treadmill exercise for 6 weeks. Cells cultured by serum from rats who did aerobic treadmill exercise for 6 weeks were separated by without NRF-2 inhibitor (EXE) and with NRF-2 inhibitor (EXE + ML). SUA level of rats was tested by using dry chemical assays, xanthine oxidase (XOD) activity in serum and liver were tested by using enzyme colorimetry assays, protein expression in kidney and NRK-52E cells were tested by using Western-blot, and UA levels in the upper or lower chamber were tested by colorimetry assays. Aerobic exercise reduced SUA levels in rats but did not significantly affect on liver xanthine oxidase. It also increased the expression of some UA transporters in the kidney and NRK-52E cells and increased the cells’ ability in UA excretion. When the NRF-2 was inhibited, the NF-κB and ABCG2 increased, and the expression of ABCC4, URAT1, and GLUT9 decreased. In conclusion, this study suggested that 6 weeks of aerobic treadmill exercise intervention may help to improve the excretion of UA in renal cells, suggesting that long-term aerobic exercise may be a means to prevent hyperuricemia.

## Introduction

UA plays an important role in kidney disease^1^. HUA is a physiological condition directly related to SUA levels. Long-term high SUA level in the body will cause a variety of tissue damage and function decline, which may induce gout^2^, kidney injury^3^, metabolic syndrome^4,5^, and other diseases that seriously affect physical health. The kidney plays a vital role in the metabolism of various substances in the body, also the main way of UA excretion in vivo. Renal urate excretion has four steps: filtration, presecretory reabsorption, secretion, and post-secretory reabsorption^6^. ABCC4, ABCG2^7,8^, GLUT9^9^, and URAT1^10^ in renal tubular epithelium play an vital role in the renal excretion of UA, and their dysfunction may affect the excretion of UA.

Exercise has multiple effects on all physiological systems of the body^11^. Regular physical activity and exercise training results in enhanced functional capacity and improved physical health, due to adaptations in tissues and organs. In recent years, it has been demonstrated that the contract of skeletal muscles could release a variety of metabolites such as bioactive proteins, which may play an important role in enhancing the functions of various tissues and organs in the body during regular exercise^12,13^. Previous studies have shown that increased metabolites in blood after exercise can improve hippocampal function^14^ and increase GLUT4 expression in human adipocytes^15^. Another research demonstrated that serum from people with long-term exercise experience also increased the protein expression of Brain-Derived Neurotrophic Factor (BDNF) and Tropomyosin-related kinase B (TrkB) of hippocampal neuronal cells (HT-22)^16^, suggesting that exercise-induced increase in circulating bioactive molecules.

Exercise could reduce SUA levels in patients with HUA and have similar effects in healthy people^17^. It has been suggested that NF-κB and NRF-2 may play an important role in regulating the expression of these UA transporters in various tissues of the body, and the effect of exercise on NF-κB and NRF-2 has also been proved^12,13^. However, the reason why exercise reduces SUA is still not apparent. This study aimed to investigate whether the serum of exercise rats affected the ability and physiological mechanism of UA transportation via ABCC4, ABCG2, URAT1, and GLUT9 of normal rat kidney cells.

## Material and method

### Animals and cells

The study was approved by the Sports Science Experimental Ethics Committee of Beijing Sport University Beijing, China (ID 2020175A). The study is reported following ARRIVE guidelines. All procedures concerning the handling and use of laboratory animals were performed following European Union (UE) regulations under Directive 2010/63/EU on the protection of animals used for scientific. Experiments were carried out following the so-called 3Rs principle (Replacement, Reduction, Refinement animals.

Twenty healthy male Sprague Dawley (SD) rats (8 weeks old, body weight 229.788 ± 16.609 g) were purchased from SiPeiFu (Beijing) Biotechnology Co. (LTD: SCXK2019-0010) were selected for this study. All rats were numbered and randomly divided into the control group (CON, n = 10) and the exercise group (EXE, n = 10) according to the random number table generated by Excel and had body weights of 230.742 ± 13.836 and 228.834 ± 19.727 g, respectively (mean ± SD). NRK-52E cell purchased from Wuhan Procell Life Science & Technology Co., LTD. (Procell, CL-0174).

### Exercise program for rats

Before the exercise intervention, rats in the EXE group were acclimatized to treadmill exercise for 3 days. After the acclimation to the treadmill, each rat was subjected to graded exercise testing on a treadmill with a 5° slope and VO_2_max was tested by using the Columbus Oxymax system (Columbus Instruments, USA). When the rat did not run after being electrical shocked, or when the result of VO_2_ decreased twice in a row, the rat was considered to have reached VO_2_max intensity. During the 6 weeks, VO_2_max was tested every two weeks, and the exercise intensity was adjusted according to the VO_2_max results. The EXE group warmed up on the treadmill for 10 min at 50% VO_2_max intensity before exercise. After the warm-up, the EXE group performed 50 min of aerobic exercise at 70% VO_2_max intensity (Slope:5°). The exercise intervention was performed for 6 weeks, 6 days per week.

### Sample collection

Twenty-four hours after the last exercise, all rats were anesthetized by intraperitoneal injection of 20% urethane solution (5 ml/kg). Blood was obtained from the rat's abdominal aorta and placed in vacuum vasculature, and serum was obtained by centrifugation. The kidney and liver of all rats were collected, and all samples were stored at − 80 °C for later use.

### Cell culture

Before cell culture, serums from rats in the same group were thoroughly mixed. NRK-52E cells were divided into control group (CON), exercise serum group (EXE), and exercise serum with ML385 group (EXE + ML) cultured in 6-well plates with DMEM + 10%FBS + 1% (Penicillin–Streptomycin Solution). After cell growth to about 80% confluence, serum-free exposure for 24 h, then exposure in a different new culture medium for 24 h (37 °C, 5% CO_2_ saturated humidity) for further tests. CON group’s medium containing 10% serum of CON rats, EXE group’s medium containing 10% serum of EXE rats, and EXE + ML group’s medium containing 10% serum of EXE rats with a high-efficiency NRF-2 inhibitor called ML385 (5 μmol/L)^18^.

### Cell uric acid transport ability

After the cells were cultured as described above for 24 h, the cells were placed in the upper chamber of ttranswell plate. The upper and lower chamber represents the apical side and basal side of kidney cells. Therefore, two different treatment methods were set for each group of cells to verify the effects of different treatment methods on UA excretion and UA reabsorption capacity of NRK-52E cells. One was treated with 300 μmol/L of UA only in the upper chamber for 24 h, and the UA level in the lower chamber was measured to reflect the effect of different intervention methods on the UA reabsorption capacity of NRK-52E cells. The other only added 300 μmol/L of UA in the lower chamber, and the effect of different intervention methods on the UA excretion ability of NRK-52E cells was reflected by measuring the UA level in the upper chamber. The UA level of upper and lower chambers was measured by colorimetric method with a UA kit (Nanjing Jiancheng Bioengineering Institute, C-012-2), aiming to reflect the UA reabsorption or excretion capacity of cells.

### Serum uric acid

Before and after the 6 weeks of exercise intervention, blood was taken from all rats through their retro-orbital plexus. Serum was obtained after centrifugation at 3500 rpm for 15 min. Add serum drops to the test strip, and SUA was measured by the dry chemical method using Wondfo DC-101(Guangzhou Wondfo Biotech Co., Ltd., CHN).

### Serum and liver XOD

Serum (obtained from rats 24 h after the last exercise) and liver XOD levels were measured by enzyme colorimetry with a rat XOD kit (Wuhan Spbio Co., LTD., CHN).

### Total Protein Extraction and Western Blot

Kidneys and NRK-52E cells were collected. The liver was homogenized at low temperature for 20 s two to three times in a precooled EP tube. After 30 min of ice bath, the supernatant was extracted after centrifugation at 4 °C for 30 min (13,000 rpm). Protein concentration in kidneys and cell lysates was determined using a BCA kit (P0010; Beyotime). The extracted protein supernatant and 5 × protein loading buffer (mixed by 4:1 volume) were placed in boiling water for 10 min. After denaturation, the proteins were cooled to indoor temperature and stored at − 20 °C Proteins (20 μL in each channel) were loaded on a pre-made gel (5% concentrated gel, 12% separated gel) and electrophoresed by DYY-7C electrophoresis apparatus (Beijing LiuYi Instrument, CHN). Gels were transferred to PVDF membranes using a CZ-40 transfer box (Beijing LiuYi Instrument, CHN), then stained with ponceau, and then cut the blots before hybridization with antibodies. Proteins were treated by immunoblotting with specific antibodies in different dilution ratios (Table 1) in TBST containing 5% skim milk powder (SMP). Then, the proteins were treated by second antibodies in TBST. Only one kind of protein and GAPDH were measured on each membrane. BandScan was used to analyze gray values.

**Table 1:** Basic information about antibodies.

	Manufacturer	Art. no	Dilution ratio
NF-κB	Santa cruz	Sc-8008	1:200
NRF-2	Santa cruz	Sc-365949	1:200
ABCG2	Abcam	Ab207732	1:1000
ABCC4	Santa cruz	Sc-59614	1:200
URAT1	Santa cruz	Sc-293405	1:200
GLUT9	Abcam	Ab223470	1:3000
HRP Conjugated AffiniPure Goat Anti-mouse IgG (H + L)	Boster	BA1051	1:10,000
HRP Conjugated AffiniPure Goat Anti-rabbit IgG (H + L)	Boster	BA1054	1:10,000
HRP Conjugated AffiniPure Rabbit Anti-rat IgG (H + L)	Boster	BA1058	1:10,000

### Statistical analysis

The SUA of all rats and the protein expression of UA transporters in NRK-52E cells were expressed in Mean ± SD. Kolmogorov–Smirnov test was performed on the obtained data using SPSS19.0, and all data were in accordance with normal distribution. SPSS 19.0 was used for the independent sample T-test for comparison between groups, and paired sample T-test for intra-group comparison of SUA, *p* < 0.05 and *p* < 0.01 were considered statistical differences.

## Result

### XOD and SUA levels

**Figure 1:**
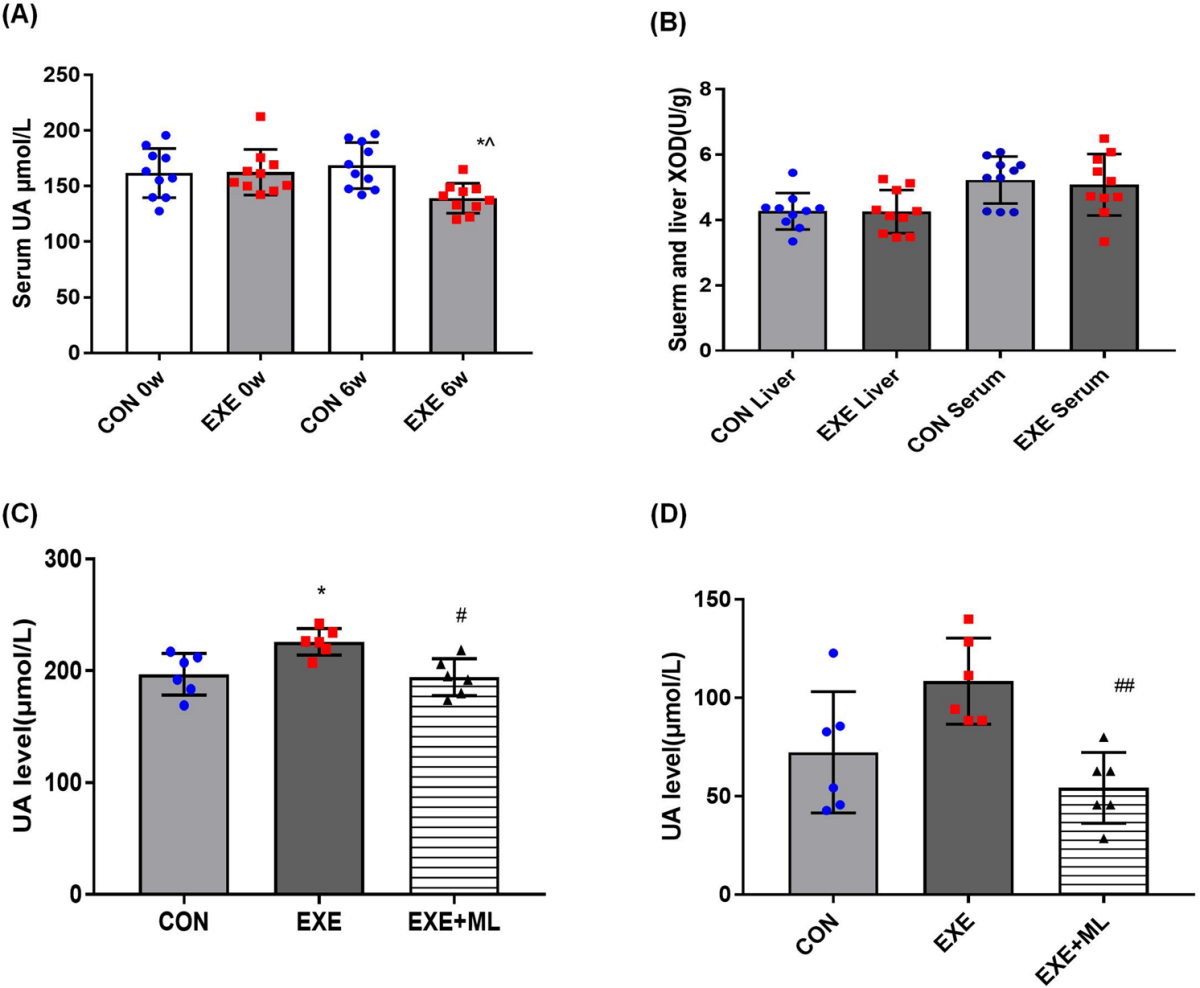
(**A**) SUA of all rats before and after 6 weeks of intervention obtained either with exercise (EXE) or without exercise (CON). (**B**) XOD level of all rats after 6 weeks of intervention in serum and liver. (**C**) UA level of the upper chamber in culture plate. (**D**) UA level of the lower chamber in culture plate. Data are means ± SD (n = 10). * Significantly different from CON (*p* < 0.05), ^ Significantly different from 0th week (*p* < 0.05), ## Significantly different from EXE (*p* < 0.01).

After 6 weeks of exercise, there was no significant difference in serum and liver XOD level between CON and EXE (Table 2 and Fig. 1B). SUA in the EXE group was lower than in the CON group (*p* < 0.05; Table 3 and Fig. 1A).

**Table 2:** Liver XOD level of all rats after 6 weeks of intervention (U/g).

	CON	EXE
Suerm XOD	5.222 ± 0.720	5.076 ± 0.939
Liver XOD	4.289 ± 0.477	4.169 ± 0.737

**Table 3:** SUA level of all rats before and after 6 weeks of intervention (μmol/L).

	CON	EXE
0th week	161.83 ± 22.12	162.71 ± 20.52
6th week	168.57 ± 20.75	139.24 ± 13.46*^

*Significantly different from CON (*p* < 0.05), ^Significantly different from 0th week (*p* < 0.05).

### UA excretion and reabsorption ability of NRK-52E cells

UA level of the upper chamber in the EXE group of cells is higher than in the CON group (*p* < 0.05; Table 4 and Fig. 1C) and the UA level of the upper chamber in the EXE + ML group of cells is lower than in the EXE group (*p* < 0.05; Table 4 and Fig. 1C). UA level of the lower chamber in the EXE + ML group of cells is lower than in the EXE group (*p* < 0.01; Table 4 and Fig. 1D).

**Table 4:** UA level in culture plate in the upper and lower chamber (μmol/L).

	CON	EXE	EXE + ML
UA level in the upper chamber	196.90 ± 18.61	225.95 ± 11.95*	194.45 ± 16.46#
UA level in the lower chamber	75.71 ± 27.41	105.24 ± 19.35	54.29 ± 17.98##

*Significantly different from CON (*p* < 0.05). #Significantly different from EXE (*p* < 0.05). ##Significantly different from EXE (*p* < 0.01).

### NF-κB and NRF-2 protein expression

**Figure 2:**
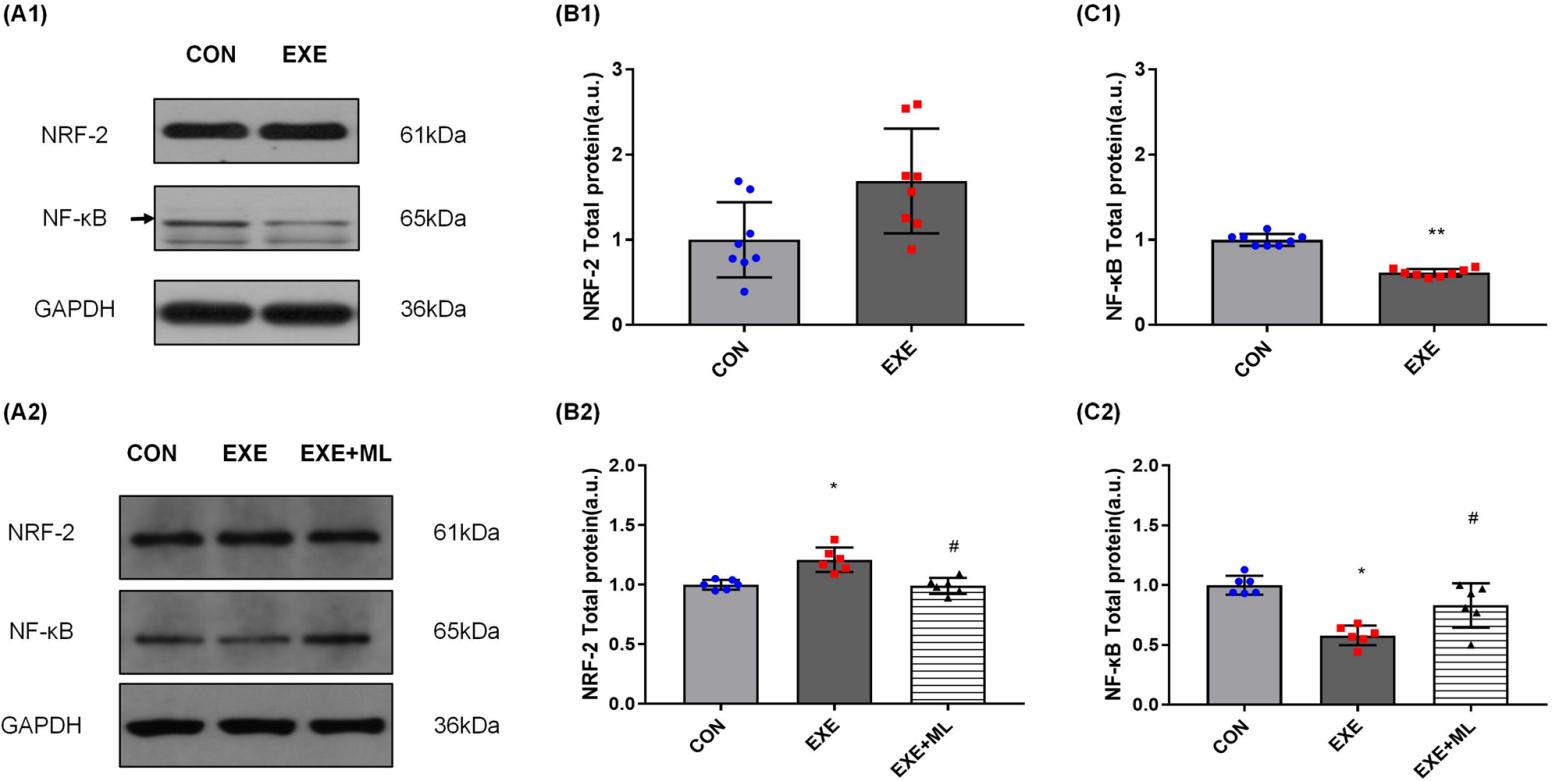
(**A1**–**C1**): NRF-2 and NF-κB protein expression, normalized against GAPDH, in rat’s kidney. Data are means ± SD (n = 8). ** Significantly different from CON (*p* < 0.01). (**A2**–**C2**): NRF-2 and NF-κB protein expression, normalized against GAPDH, in NRK-52E cells 24 h after exposure to 10% serum without exercise (CON), serum with exercise (EXE) or serum with exercise supplemented with ML385. Data are means ± SD (n = 6). * Significantly different from CON (*p* < 0.05), # Significantly different from EXE (*p* < 0.05).

After 6 weeks of exercise, NRF-2 expression in the EXE group was higher than in the CON group, but there was no significant difference in NRF-2 expression between the CON and EXE groups of rats (Table 5 and Fig. 2A1, B1). The expression of NF-κB in the kidneys of the EXE group was significantly lower than in the CON group (*p* < 0.01; Table 5 and Fig. 2A1, C1).

**Table 5:** NF-κB and NRF-2 protein expression of rat’s kidney.

	CON	EXE
NRF-2	1.000 ± 0.168	1.158 ± 0.166
NF-κB	1.000 ± 0.068	0.613 ± 0.044**

**Significantly different from CON (*p* < 0.01).

Exposure of NRK-52E cells to 10% serum obtained after 6 weeks of exercise for 24 h increased NRF-2 protein expression, on average, by 21.0% (*p* < 0.01; Table 6 and Fig. 2A2, B2), compared with exposure to 10% serum obtained from rats who did not exercise. Exposure of NRK-52E cells to 10% serum obtained after 6 weeks of exercise with ML385 for 24 h decreased NRF-2 protein expression (*p* < 0.01; Table 6 and Fig. 2A2, B2), compared with exposure to 10% serum obtained after exercise but without ML385.

**Table 6:** NF-κB and NRF-2 protein expression of NRK-52E cell.

	CON	EXE	EXE + ML
NRF-2	1.000 ± 0.043	1.210 ± 0.104*	0.989 ± 0.069#
NF-κB	1.000 ± 0.079	0.578 ± 0.084*	0.830 ± 0.187#

*Significantly different from CON (*p* < 0.05), #Significantly different from EXE (*p* < 0.05).

Exposure of NRK-52E cells to 10% serum obtained after 6 weeks of exercise for 24 h decreased NF-κB protein expression, on average, by 44.2% (*p* < 0.01; Table 6 and Fig. 2A2, C2), compared with exposure to 10% serum obtained not exercise. Exposure of NRK-52E cells to 10% serum obtained after 6 weeks of exercise with ML385 for 24 h increased NF-κB protein expression (*p* < 0.05; Table 6 and Fig. 2A2, C2), compared with exposure to 10% serum obtained after exercise but without ML385.

### ABCC4 and ABCG2 protein expression

**Figure 3:**
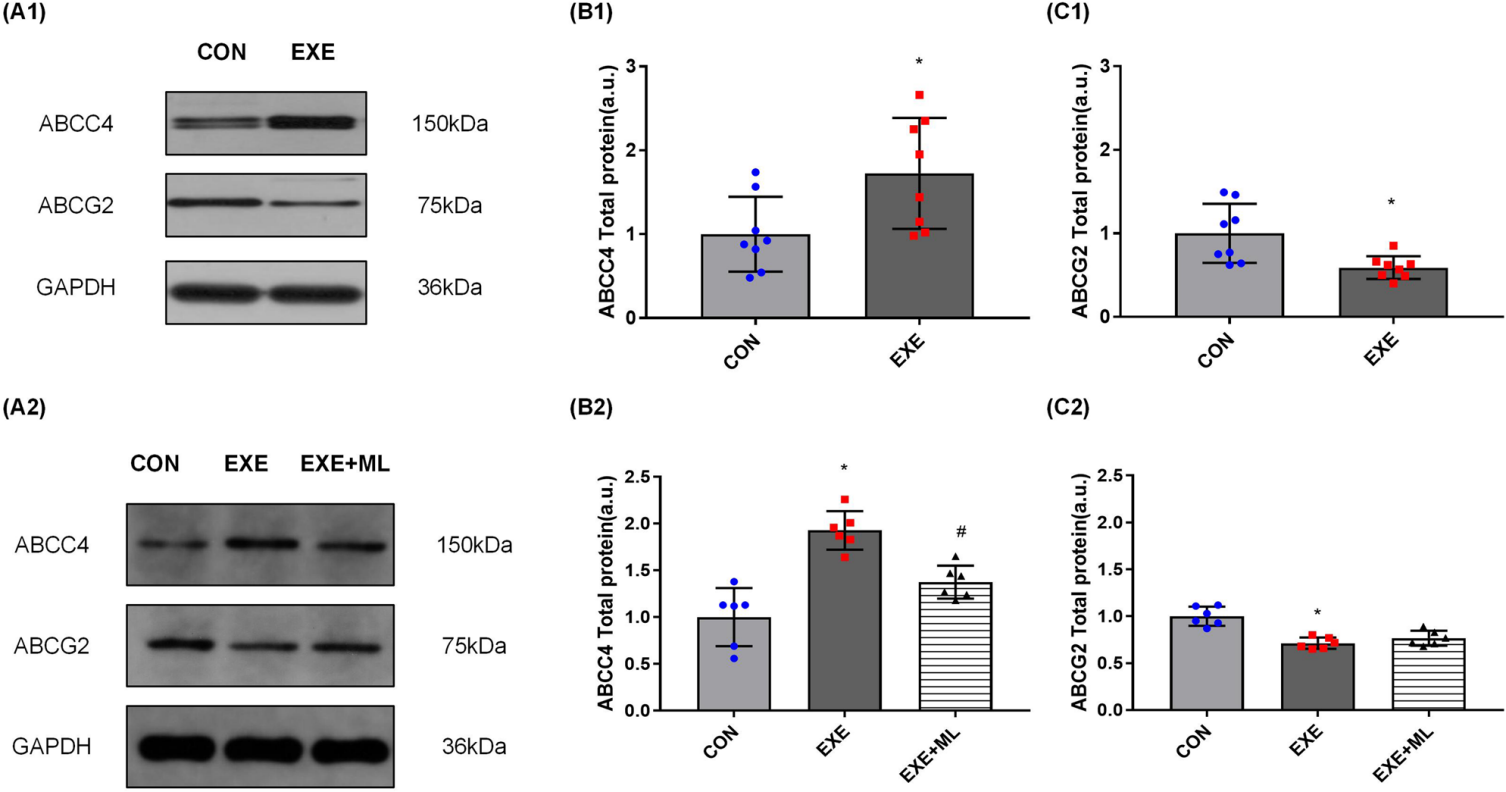
(**A1**–**C1**): ABCC4 and ABCG2 protein expression, normalized against GAPDH, in rat’s kidney. Data are means ± SD (n = 8). * Significantly different from CON (*p* < 0.05). (**A2**–**C2**): ABCC4 and ABCG2 protein expression, normalized against GAPDH, in NRK-52E cells 24 h after exposure to 10% serum without exercise (CON), serum with exercise (EXE) or serum with exercise supplemented with ML385. Data are means ± SD (n = 6). * Significantly different from CON (*p* < 0.05), # Significantly different from EXE (*p* < 0.05).

After 6 weeks of exercise, the expression of ABCC4 in the kidneys of the EXE group was significantly higher than the CON group (*p* < 0.05; Table 7 and Fig. 3A1, B1). The expression of ABCG2 in the kidneys of the EXE group was significantly lower than the CON group (*p* < 0.05; Table 7 and Fig. 3A1, C1).

**Table 7:** ABCC4 and ABCG2 protein expression of rat’s kidney.

	CON	EXE
ABCC4	1.000 ± 0.447	1.726 ± 0.662*
ABCG2	1.000 ± 0.355	0.591 ± 0.138*

*Significantly different from CON (*p* < 0.05).

Exposure of NRK-52E cells to 10% serum obtained after 6 weeks of exercise for 24 h increased ABCC4 protein expression, on average, by 92.7% (*p* < 0.01; Table 8 and Fig. 3A2, B2) and decreased ABCG2 protein expression, on average, by 28.9% (*p* < 0.01; Table 8 and Fig. 3A2, C2).Exposure of NRK-52E cells to 10% serum obtained after 6 weeks of exercise with ML385 for 24 h decreased ABCC4 protein expression (*p* < 0.01; Table 8 and Fig. 3 A2, B2) compared with exposure to 10% serum obtained after exercise but without ML385.

**Table 8:** ABCC4 and ABCG2 protein expression of NRK-52E cell.

	CON	EXE	EXE + ML
ABCC4	1.000 ± 0.310	1.927 ± 0.207*	1.375 ± 0.178#
ABCG2	1.000 ± 0.103	0.711 ± 0.062*	0.768 ± 0.080

*Significantly different from CON (*p* < 0.05), #Significantly different from EXE (*p* < 0.05).

### URAT1 and GLUT9 protein expression

**Figure 4:**
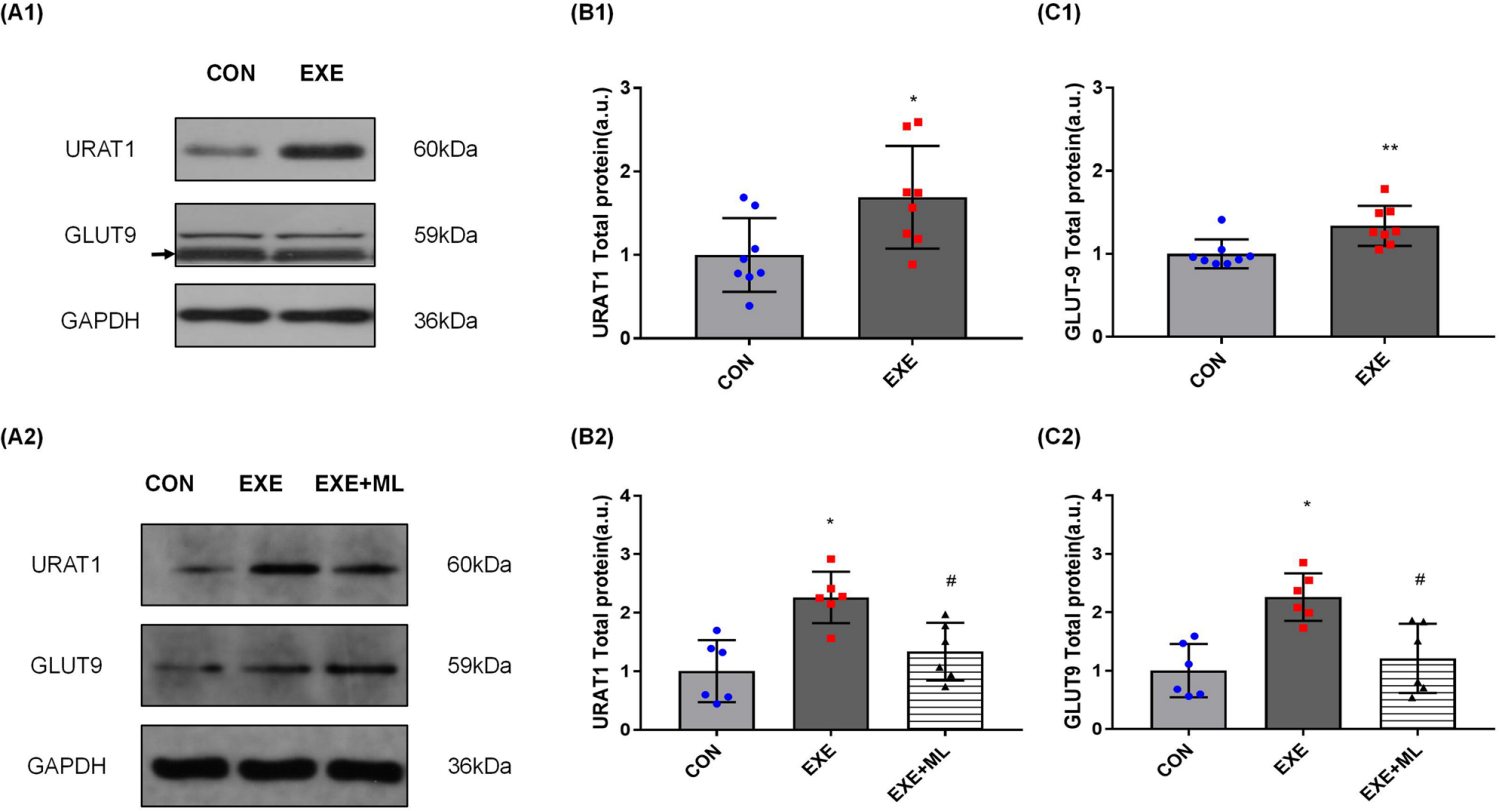
(**A1**–**C1**): ABCC4 and ABCG2 protein expression, normalized against GAPDH, in rat’s kidney. Data are means ± SD (n = 8). * Significantly different from CON (*p* < 0.05), ** Significantly different from CON (*p* < 0.01). (**A2**–**C2**): URAT1 and GLUT9 protein expression, normalized against GAPDH, in NRK-52E cells 24 h after exposure to 10% serum without exercise (CON), serum with exercise (EXE) or serum with exercise supplemented with ML385. Data are means ± SD (n = 6). * Significantly different from CON (*p* < 0.05), # Significantly different from EXE (*p* < 0.05).

After 6 weeks of exercise, the expression of URAT1 in the kidneys of the EXE group was significantly higher than the CON group (1.691 ± 0.614 vs. 1.000 ± 0.442 a.u., *p* < 0.05; Table 9 and Fig. 4A1, B1). The expression of GLUT9 in the kidneys of the EXE group was significantly higher than the CON group (1.338 ± 0.242 vs. 1.000 ± 0.173 a.u., *p* < 0.05; Table 9 and Fig. 4A1, C1).

**Table 9:** URAT1 and GLUT9 protein expression of rat’s kidney.

	CON	EXE
URAT1	1.000 ± 0.442	1.691 ± 0.614
GLUT9	1.000 ± 0.173	1.338 ± 0.242

*Significantly different from CON (*p* < 0.05).

Exposure of NRK-5he2E cells to 10% serum obtained after 6 weeks of exercise for 24 h increased URAT1 protein expression, on average, by 126.2% (2.262 ± 0.441 vs. 1.000 ± 0.531 a.u. *p* < 0.01; Table 10 and Fig. 4A2, B2), compared with exposure to 10% serum obtained not exercise. Exposure of NRK-52E cells to 10% serum obtained after 6 weeks of exercise with ML385 for 24 h decreased URAT1 protein expression (1.337 ± 0.494 vs. 2.262 ± 0.441 a.u., *p* < 0.01; Table 10 and Fig. 4A2, B2), compared with exposure to 10% serum obtained after exercise but without ML385.

**Table 10:** URAT1 and GLUT9 protein expression of NRK-52E cell.

	CON	EXE	EXE + ML
URAT1	1.000 ± 0.531	2.262 ± 0.441*	1.337 ± 0.494#
GLUT9	1.000 ± 0.550	2.260 ± 0.408*	1.212 ± 0.595#

*Significantly different from CON (*p* < 0.05), #Significantly different from EXE (*p* < 0.05).

Exposure of NRK-52E cells to 10% serum obtained after 6 weeks of exercise for 24 h increased GLUT9 protein expression, on average, by 126.0% (2.260 ± 0.409 vs. 1.000 ± 0.550 a.u. *p* < 0.01; Table 10 and Fig. 4A2, C2), compared with exposure to 10% serum obtained not exercise. Exposure of NRK-52E cells to 10% serum obtained after 6 weeks of exercise with ML385 for 24 h decreased GLUT9 protein expression (1.212 ± 0.595 vs. 2.260 ± 0.409 a.u., *p* < 0.01; Table 10 and Fig. 4A2, C2), compared with exposure to 10% serum obtained after exercise but without ML385.

## Discussion

In the downstream pathway of purine metabolism, XOD not only catalyzed the metabolism of hypoxanthine from intermediate to xanthine and then to UA, but also directly catalyzed the synthesis of xanthine from intermediate to UA, which was a key rate-limiting enzyme in the production of UA. The increase of XOD activity was one of the main reasons of HUA^19^. Our study showed no significant difference in XOD activity in rat liver after 6 weeks of aerobic exercise, suggesting that 6 weeks of aerobic exercise did not affect on UA production in rats. After 6 weeks of aerobic exercise, the SUA level of the rats was lower than that of the control rats.

When the cells were cultured in transwell plate, the upper and lower chamber represented the apical side and basal side of kidney cells. Therefore, the transport of UA in the lower chamber to the upper chamber depends on the UA excretion function of cells. In comparison the transport of UA in the upper chamber to the lower chamber depends on the UA reabsorption function of cells. Circulating factors in the blood after long-term exercise increased UA level in the upper chamber in NRK-52E cells after 24 h of serum exposure. When NRF-2 was inhibited, the UA level in the upper chamber was reduced. These results suggest that the decrease of SUA in rats is related to the increase of renal UA excretion, and the function of UA transporters in the cells may be related to the function of NRF-2.

NF-κB and NRF-2 play a vital role in regulating of inflammatory response^20,21^ and oxidative stress^22^. In addition, the functions of some UA transporters are regulated by NF-κB and NRF-2 has been confirmed^23–25^. These suggested that NF-κB and NRF-2 play an vital role in HUA’s pathogenesis and clinicopathological phenotype. Long-term regular aerobic exercise can also improve the body’s antioxidant capacity by regulating the expression of NF-κB and NRF-2 in the kidney^26^, weaken oxidative stress injury, reduce the inflammatory response, alleviate kidney injury, and improve kidney function^27,28^. In this study, after 6 weeks of aerobic exercise, the expression of NF-κB in the EXE group of rats was significantly lower than in the CON. Although NRF-2 expression was not significantly different between the EXE and CON groups of rats, most of the individuals in the EXE group had a higher level of NRF-2 expression than in the CON group. Circulating factors in the blood after long-term exercise increased NRF-2 expression and decreased NF-κB expression in NRK-52E cells after 24 h of serum exposure. These results suggest that 6 weeks of aerobic exercise can improve the antioxidant capacity of the kidney and cells, inhibit the inflammatory response and protect the kidney.

ABCC4 (MRP4) is an important pathway for the excretion of UA by renal cells^29^. It drains UA into the tubule through epithelial cells by consuming ATP so that UA can be excreted with urine^30^. When the expression of ABCC4 is inhibited, the UA excretion by the kidney is reduced, which is a risk for HUA and gout^31^. ABCG2 regulates the secretion of UA in the anterior membrane of renal tubular epithelial cells by consuming ATP^32,33^. When the ABCG2 protein expression increased, the excretion of UA increase^34,35^, confirming that ABCG2 plays an vital role in renal UA excretion, and the functional changes of ABCG2 are also closely related to the risk of HUA^36^.

ABCC4 and ABCG2 have the function of excreting UA in the kidney and serve as substrates for the transport of various anti-cancer drugs and environmental carcinogens in various organs^37,38^. Therefore, current studies mainly focus on the effects of different medications or environments on ABCC4 and ABCG2 functions, and relevant research demonstrated that drugs with an antioxidant effects might increase the expression of ABCC4 and ABCG2 by regulating the expression of NRF-2 and NF-κB^24^. Aerobic exercise also has antioxidant effect, and its positive effect on NRF-2 expression in various tissues and organs of the body has been demonstrated, suggesting that circulating factors in serum may have positive effects on ABCC4 and ABCG2 expression through NRF-2 pathway after long-term aerobic exercise^39,40^. However, few studies have focused on the effects of exercise on ABCC4 and ABCG2 expression. Only one dissertation reported that ABCG2 expression increased in the hippocampus of rats after a period of treadmill exercise^41^, the effect of exercise on ABCC4 and ABCG2 expression in the kidney has not been reported yet.

Our results suggest that aerobic exercise increased ABCC4 expression in healthy rats’ kidneys, and circulating factors in the blood after long-term exercise increased ABCC4 expression in NRK-52E cells after 24 h of serum exposure. This result suggested that the expression changes of UA transporters in the kidney after exercise are related to circulating factors in the blood. But when the NRF-2 was inhibited in the cell, the expression of ABCC4 declined. These results indicate that circulating factors in serum after long-term exercise may significantly improve the ability of renal tubular epithelium to excrete UA via ABCC4, and the expression of ABCC4 is positively correlated with the expression of NRF-2. This phenomenon is the same as the variation trend of proteins in other tissues in previous studies^39,42^, which is the same as the hypothesis of our study. But in this study, the expression of ABCG2 decreased in rats’ kidneys after 6 weeks of aerobic exercise, and the expression of ABCG2 decreased in NRK-52E cells after 24 h of serum exposure. When the NRF-2 was inhibited, the expression of NF-κB and ABCG2 increased, suggesting that long-term exercise may weaken the ability of renal tubular epithelium to excrete UA via ABCG2. Although the same trend in ABCG2 and NF-κB was consistent with our hypothesis and previous studies, the decline of ABCG2 in cells exposed to serum after exercise was completely contrary to our hypothesis. Our study found that the changes in NF-κB and NRF-2 were opposite. These results suggested that aerobic exercise could not activate the expression of NF-κB and NRF-2 at the same time. The main reason for ABCG2 inhibition may be related to the decreased expression of NF-κB after exercise. The expression of NF-κB and ABCG2 was increased in NRK-52E cells (EXE + ML group) after ML385 inhibition of NRF-2, which also confirmed the above view.

Moreover, relevant studies have shown that ABCG2 expression depends on the level of intracellular UA^43^, so it is speculated that the decreased expression of ABCG2 after serum exposure may be related to the low concentration of UA in the culture medium. Therefore, further research is needed to explore the causes of these phenomena.

URAT1 is a UA transporter expressed in the brush margin of proximal convoluted tubules in the renal cortex, which can reabsorb UA in about half of the proximal convoluted tubules^44^. One relevant study suggested that mice’s renal UA reabsorption function was decreased by knockout of URAT1, which confirmed the reabsorption effect on UA of URAT1^10^. Our results indicate that aerobic exercise increased URAT1 expression in healthy rats’ kidneys, and circulating factors in the blood after long-term exercise increased URAT1 expression in NRK-52E cells after 24 h of serum exposure. When the NRF-2 was inhibited, the expression of URAT1 declined. This result indicates that the expression of URAT1 was positively correlated with NRF2, and circulating factors in serum after long-term exercise may significantly improve the ability of renal tubular epithelium to resorb UA via URAT1. However, the expression of URAT1 increased is not consistent with our research hypothesis that long-term aerobic exercise decreases SUA by increasing UA's excretion and decreasing UA's reabsorption via renal tubular epithelium. Relevant studies suggested that UA is an antioxidant in the body, and the balance of SUA levels is crucial to maintaining the body’s antioxidant capacity^45^. In this study, the elevation of URAT1 expression in kidneys and cells caused by circulating factors in blood after 6 weeks of aerobic exercise may be a protective mechanism to prevent the elevation of other UA transporters from causing excessive UA excretion. Studies showed that URAT1 expression in HUA individuals was higher than in healthy individuals^46,47^. URAT1 protein activity increased after exercise in healthy rats^6^, and this study proposed that exercise-induced changes in URAT1 expression and activity were associated with increased blood urate levels. However, our results suggest that changes in URAT1 expression after exercise are not solely related to SUA levels. Moreover, the expression of URAT1 was also associated with hypouricemia in addition to HUA^48^. The increase of URAT1 expression of cells in our research may be related to the low UA environment, so it is speculated that the increased expression of URAT1 after serum exposure in our study may be associated with the low concentration of UA in the culture medium. However, further experiments still need to explore the reason for this phenomenon.

GLUT9 is mainly expressed in the liver and kidney^49–52^. Although it is named glucose transporter 9, the main transport capacity of GLUT9 is reflected in the transport of UA rather than glucose^9^. One relevant study showed that when the expression of GLUT9 reduced, the SUA level reduced in vivo^46^, which confirmed the reabsorption effect on UA of GLUT9^48^. Our results suggest that aerobic exercise increased GLUT9 expression in healthy rat kidneys, and circulating factors in the blood after long-term exercise increased GLUT9 expression in NRK-52E cells after 24 h of serum exposure. When the NRF-2 was inhibited, the expression of GLUT9 declined. This result indicates that the expression of GLUT9 was also positively correlated with NRF2, and circulating factors in serum after long-term exercise may greatly improve the ability of renal tubular epithelium to reabsorb UA via GLUT9.

In conclusion, circulation factors in blood after long-term aerobic exercise play an important role in regulating the expression of renal UA transporters, and circulation factors in blood regulate ABCC4, URAT1, and GLUT9 by regulating NRF-2, and ABCG2 by regulating NF-κB. It is unclear which circulating factor changes in serum after exercise ultimately affect NRF-2 and NF-κB. Our results showed that long-term aerobic exercise could promote the UA excretion ability of the kidney and reduce the SUA level in a healthy body, suggesting that aerobic exercise may be a means of HUA prevention. It also provides a theoretical basis for studying the physiological mechanism of exercise prevention of HUA in the future.

## Supplementary Information

Supplementary Information.

## Data Availability

The raw data supporting the conclusions of this article will be made available by the authors, without undue reservation. If you need data from this article, you could contact with Zhongye Jiang (1210962636@qq.com) or Prof. Jianmin Cao (BB45_Colorado@foxmail.com) by e-mail.
