# Supplementary material for: Exercise serum regulates uric acid transporters in normal rat kidney cells
Source: Sci Rep. 2022 Oct 27;12:18086. doi: 10.1038/s41598-022-22570-w (PMC9613886; doi:10.1038/s41598-022-22570-w)

WB results of rat kidney

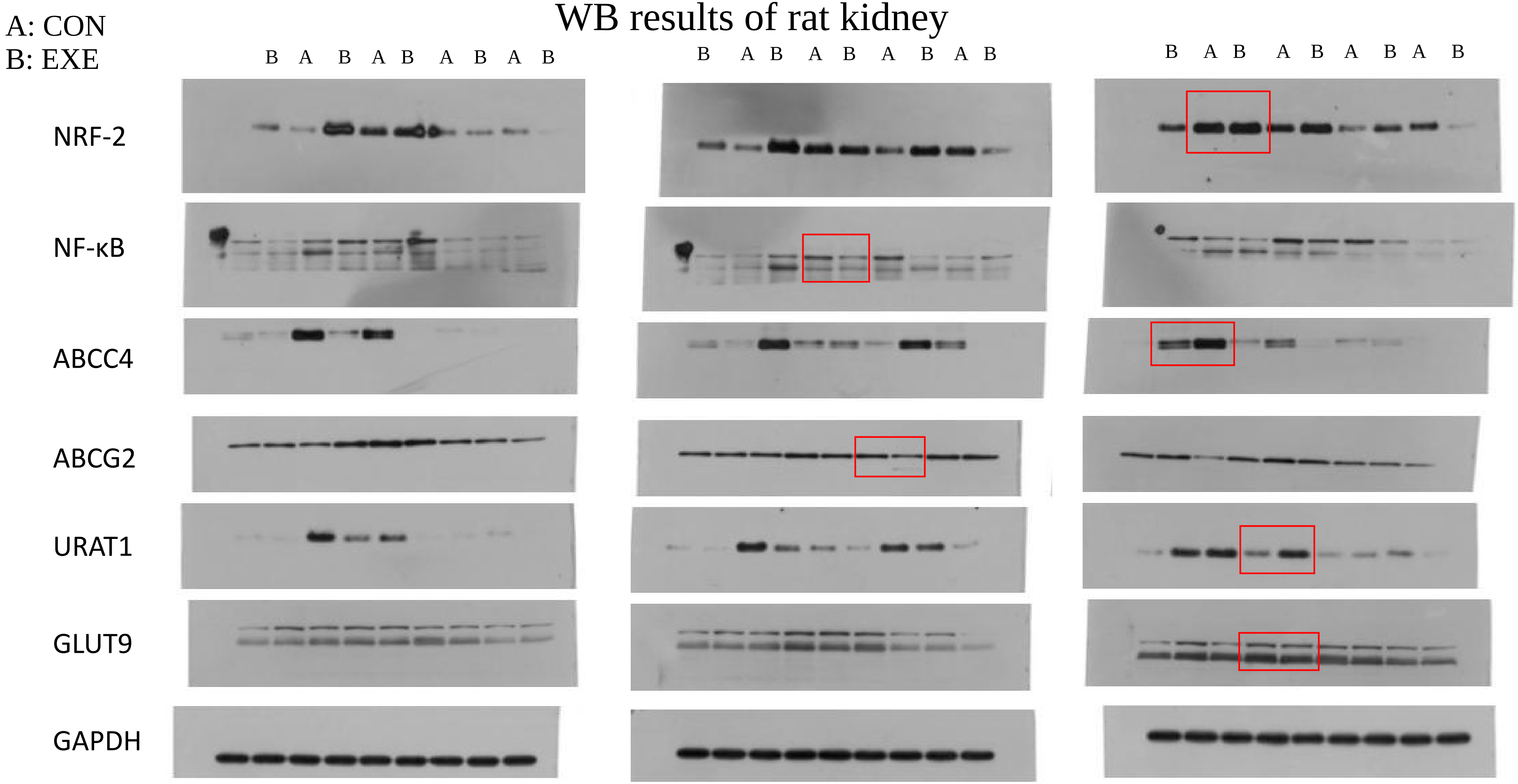

A:CON
B:EXE
C:EXE+ML

WB results of NRK-52E cells

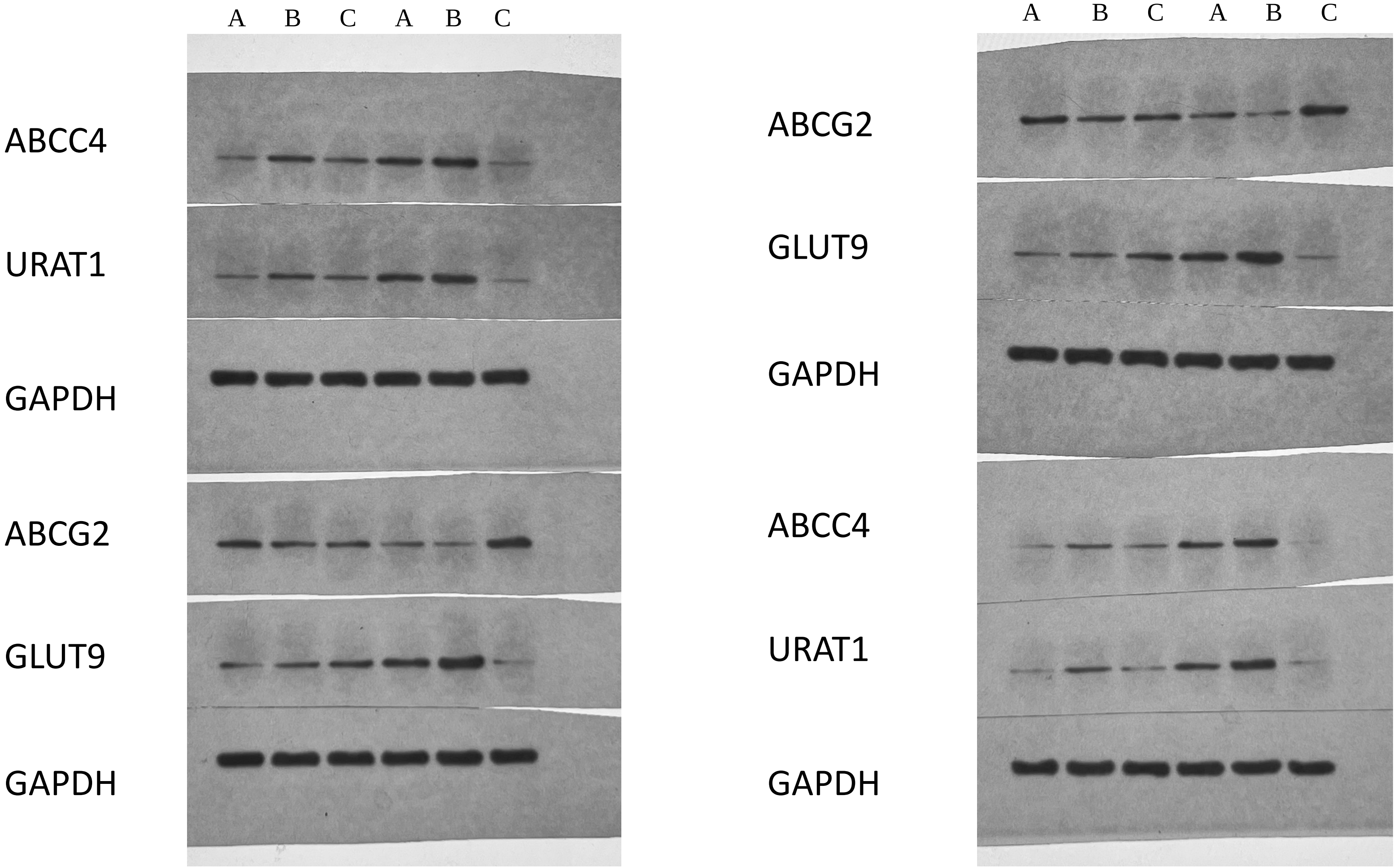

WB results of NRK-52E cells

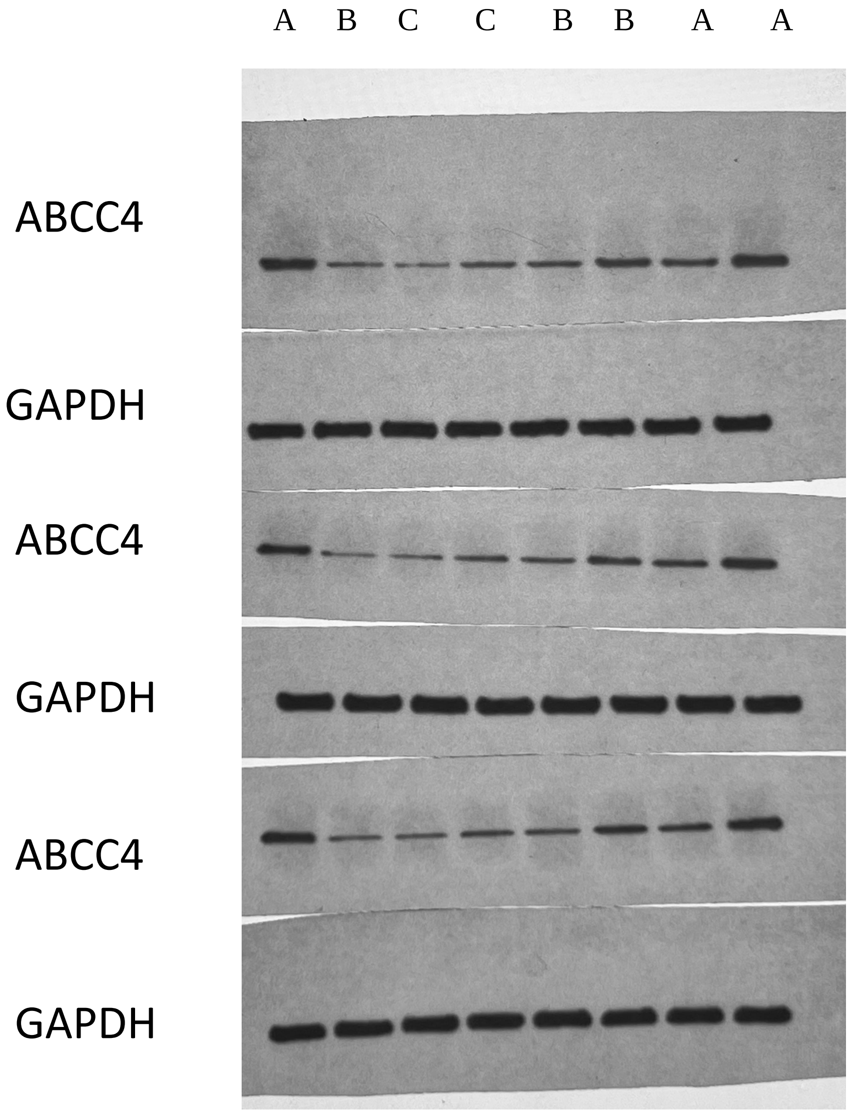

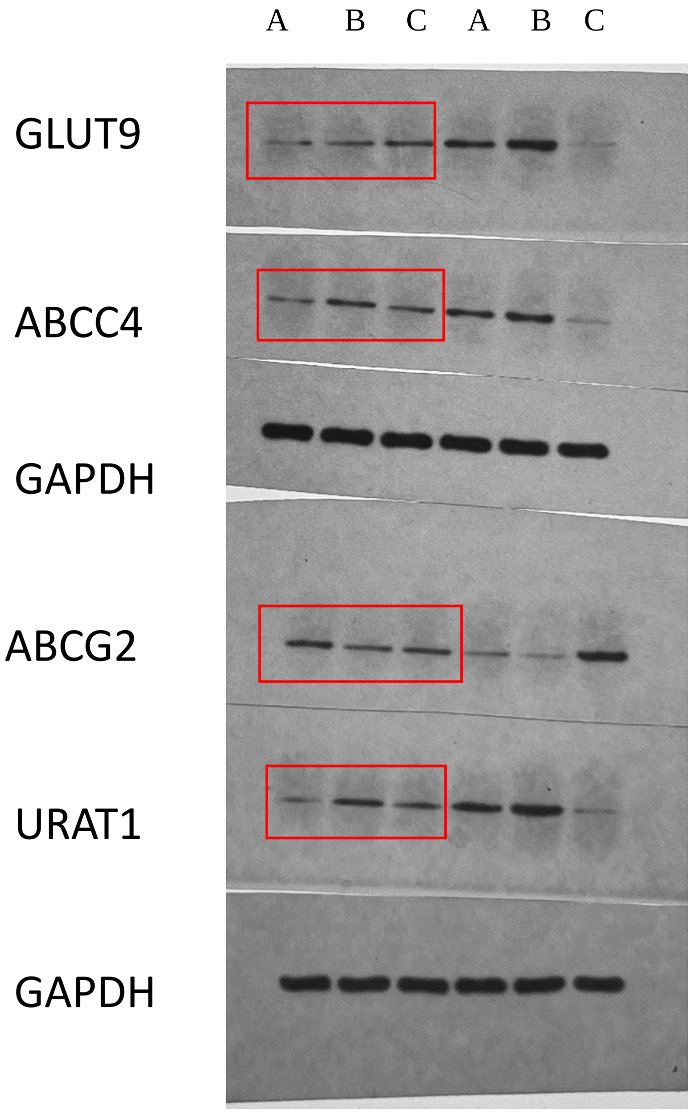

WB results of NRK-52E cells

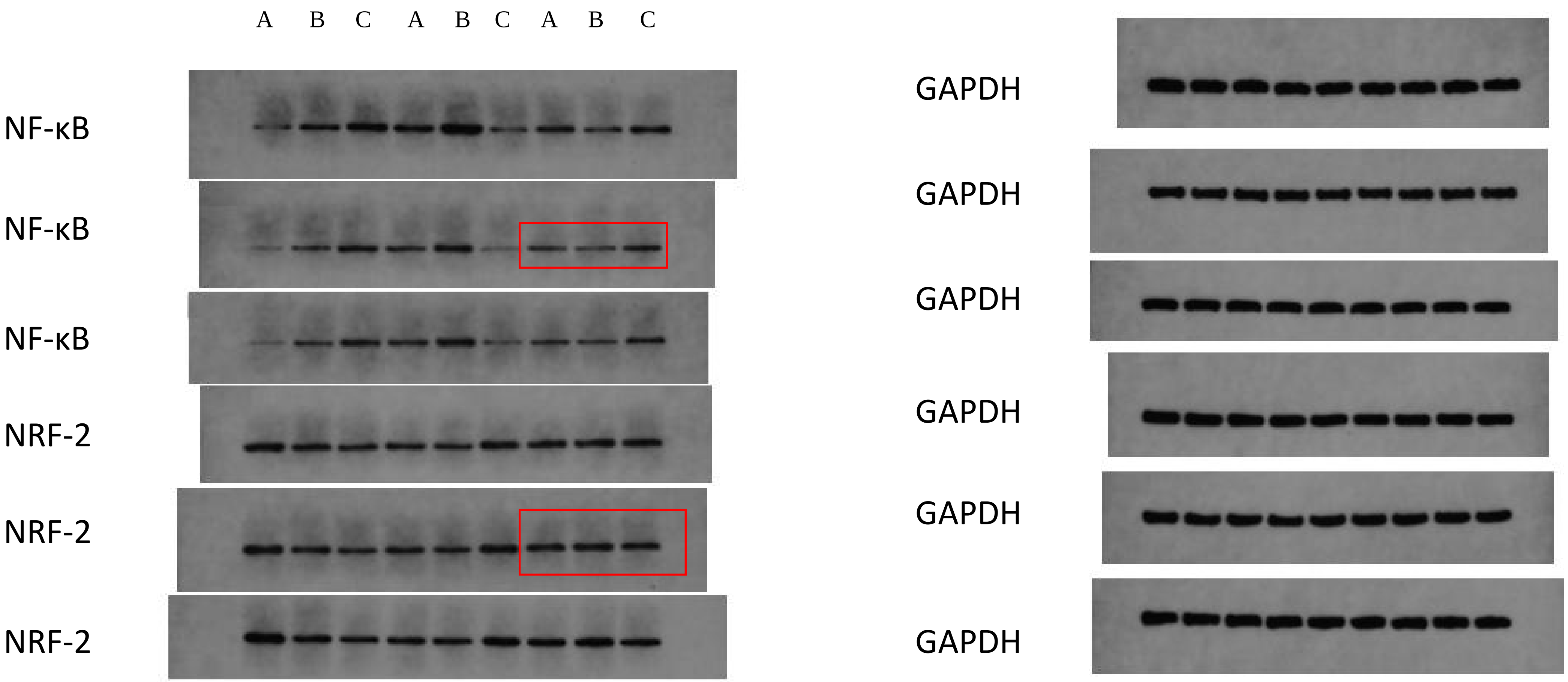

Supplement: Supplementary file 1 — Supplementary Information. [file 41598_2022_22570_MOESM1_ESM.pdf]
